# GLI1 Is a Central Mediator of EWS/FLI1 Signaling in Ewing Tumors

**DOI:** 10.1371/journal.pone.0007608

**Published:** 2009-10-27

**Authors:** Jay Joo, Laura Christensen, Kegan Warner, Leith States, Hyung-Gyoo Kang, Kieuhoa Vo, Elizabeth R. Lawlor, William A. May

**Affiliations:** 1 Division of Hematology-Oncology, Department of Pediatrics, Childrens Hospital Los Angeles and the Saban Research Institute, University of Southern California Los Angeles, Los Angeles, California, United States of America; 2 Department of Pathology, Childrens Hospital Los Angeles and the Saban Research Institute, University of Southern California Los Angeles, Los Angeles, California, United States of America; Institute of Cancer Research, United Kingdom

## Abstract

The Ewing Sarcoma Family Tumors (ESFT) consist of the classical pathologic entities of Ewing Sarcoma and peripheral Primitive Neuroectodermal Tumor. Occurring largely in the childhood through young adult years, these tumors have an unsurpassed propensity for metastasis and have no defined cell of origin. The biology of these aggressive malignancies centers around EWS/FLI1 and related EWS/ETS chimeric transcription factors, which are largely limited to this tumor class. Much progress has been made in the identification of a network of loci whose expression is modulated by EWS/FLI1 and its congeners. To date, little progress has been made in reconstructing the sequence of direct and indirect events that produce this network of modulated loci. The recent identification of GLI1 as an upregulated target of EWS/ETS transcription factors suggests a target which may be a more central mediator in the ESFT signaling network. In this paper, we further define the relationship of EWS/FLI1 expression and GLI1 upregulation in ESFT. This relationship is supported with data from primary tumor specimens. It is consistently observed across multiple ESFT cell lines and with multiple means of EWS/FLI1 inhibition. GLI1 inhibition affects tumor cell line phenotype whether shRNA or endogenous or pharmacologic inhibitors are employed. As is seen in model transformation systems, GLI1 upregulation by EWS/FLI1 appears to be independent of Hedgehog stimulation. Consistent with a more central role in ESFT pathogenesis, several known EWS/FLI1 targets appear to be targeted through GLI1. These findings further establish a central role for GLI1 in the pathogenesis of Ewing Tumors.

## Introduction

Much of the unique biology of the Ewing Sarcoma Family Tumors (ESFT) stems from the unique effects of EWS/FLI1. This fusion transcription factor, along with related EWS/ETS fusions, is virtually pathognomonic of these aggressive malignancies[1]. Given the nature of these chimeric proteins, considerable work has gone into the identification of the transcriptional targets of EWS/FLI1[2], [3]. Despite this effort, no identified target has been clinically demonstrated to be of prognostic or therapeutic significance. Together, this diverse group of targets constitute a signaling network. Elements of this transcriptional network have been identified[3] but the relationship between these elements has not been well studied. In a sense, such relationships constitute the topology of this network. Based on the biology of this disease, one can presume that EWS/FLI1 will be central to this network. But targets of EWS/FLI1 will vary in importance from isolated clients on the network to more centrally situated hubs or routers which regulate a subdomain of this network in concert. Establishing the existence and nature of such relationships will be critical to prioritizing which transcriptional targets are most likely to have maximal impact as targets for translational therapeutics.

The recent finding that EWS/FLI1 enhances expression of GLI1 presents a potential clue to the interpretation of this network[4], [5]. GLI1 is the principal transcriptional effector of the Hedgehog-GLI (HH-GLI) signaling pathway[6]. This pathway is of critical importance in many developmental processes and is important in the maintenance of stem cell compartments in both developing and mature tissues[7]. Furthermore, HH-GLI has been found to be involved in many human cancers from prostate cancer in adults to childhood medulloblastoma[8]. Translational efforts to target this pathway are ongoing[9], [10], [11]. While it has been implicated in EWS/FLI1 biology, much of this data comes from a murine model system for EWS/FLI1 transformation[4]. The establishment of the significance of GLI1 upregulation to ESFT biology remains to be more firmly established. Beyond this, if GLI1 is more than a peripheral event in the EWS/FLI1 signaling network, it can be expected to to leave an identifiable transcriptional footprint which may encompass some previously identified EWS/FLI1 targets.

Here we demonstrate that ESFT primary tumors express HH-GLI pathway members in a manner consistent with that seen in model transformation systems. The EWS/FLI1 dependence of GLI1 expression and signaling in multiple ESFT cell lines is clearly demonstrated. Using multiple means of GLI1 inhibition, we demonstrate the importance of GLI1 to the ESFT tumorigenic phenotype. Intriguingly, we show that GLI1 upregulation in ESFT is a Hedgehog independent phenomenon in ESFT, suggesting non-canonical mechanism of pathway activation. Finally, in multiple ESFT cell lines, we demonstrate that several loci known to be transcriptionally modulated by EWS/FLI1 are dependent upon GLI1 expression. This establishes GLI1 as a higher order target in the EWS/FLI1 signaling network and begins to define a hierarchy in the EWS/FLI1 signaling network.

## Results

### Primary tumors demonstrate significant GLI1 expression

**Figure 1:**
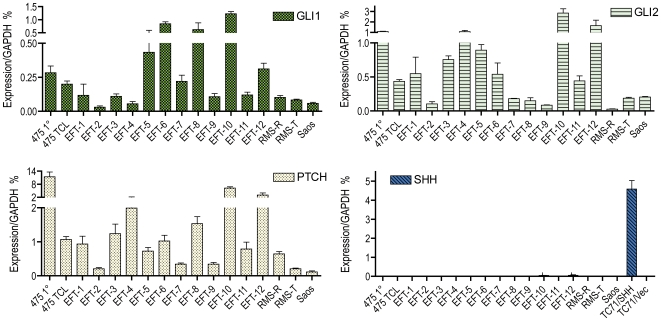
Expression of HH-GLI pathway species in primary ESFT specimens. Twelve CHTN banked tumor specimens are assessed for HH-GLI1 related gene expression by real time quantitative PCR. Also included is TTC475 in both primary tumor specimen and cell line form. For comparison, two Rhabdomyosarcoma (RMS-R and -T) and an Osteosarcoma (Saos) cell line are included. SaOS2 is in the upper quartile for GLI1 expression in NCI60 microarray data. For reference, TC71 cells transduced with SHH or empty retroviral vector are included in the SHH panel.

Our earlier findings focused on EWS/FLI1 activation of GLI1 in an NIH3T3 model transformation system[4] with added data from ESFT cell lines. However, HH-GLI pathway activity has been found to be diminished in in vitro cultured medulloblastoma lines[12], so the cell lines we evaluated may not reflect the condition in primary ESFT. To see how well these findings apply to clinical disease, we evaluated the status of a panel of 12 ESFT primary tumor specimens. As is illustrated in Figure 1, the expression of mediators of the HH-GLI pathway closely resembles that found in EWS/FLI1 expressing NIH3T3 cells. The most characteristic indicators of oncogenic signaling via this pathway are the expression levels of GLI1, GLI2 and the direct GLI1 target Patched1. These are important components of what has been termed the GLI code[13]. In these twelve ESFT specimens, we found expression levels of these pathway mediators to be similar or higher than those in specimens from cell lines known to be in the upper quartile for expression in microarray data obtained for the NCI-60 panel of tumor cell lines (Novartis, http://wombat.gnf.org). Our earlier data from NIH3T3 indicated little or no expression of Sonic Hedgehog (SHH)[4], which mediates pathway activity in development and in some tumor systems. Once again, our primary tumor specimens demonstrate very little to no expression of SHH transcript when compared to a positive control expressing SHH cDNA. Similar low levels of Indian Hedgehog (IHH) were also found (data not shown). Some of the variability of expression of GLI pathway members from sample to sample could reflect variable tumor cell content in the primary specimens. All specimens showed either an EWS/FLI1 or an EWS/ERG transcript by RT-PCR, indicating at least some ESFT tumor cell content. However the percentage of the specimen which was tumor cells could not be assessed. Another source of variability could relate to some of the other signaling pathways such as PI3-AKT, RAS-MEK1, and PKC-delta which have been shown to affect signaling in the HH-GLI1 pathway[14]. Nevertheless, overall these findings indicate that primary ESFT demonstrate significant expression of HH-GLI1 mediators and appear to demonstrate little expression of Hedgehog species.

### Multiple means of inhibition demonstrate GLI1 to be EWS/FLI1 dependent in ESFT

**Figure 2:**
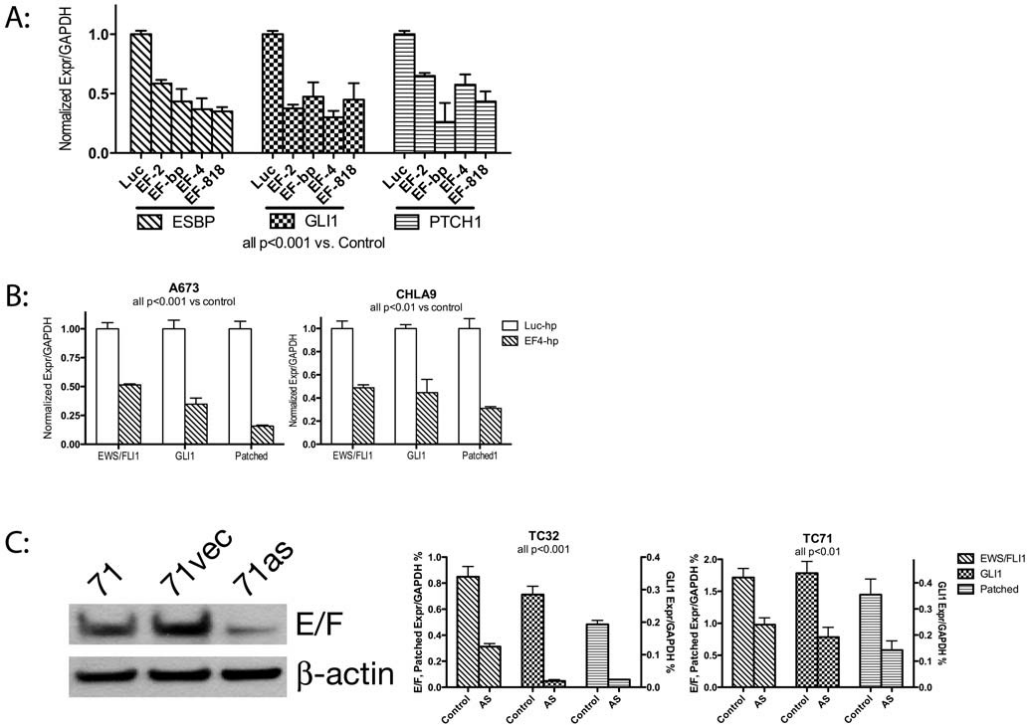
RNAi mediated reduction in EWS/FLI1 expression results in a consistent reduction of GLI1 signaling. Panel A: Four different shRNA sequences targeting EWS/FLI1 were introduced into the Ewing cell line TC71. The sequences are EF-2[26], EF-bp (from the Type 1 EWS/FLI1 breakpoint)[34], EF-4 [26] and EF-818 [33]. These are compared to a non-targeting control shRNA directed to a sequence in the luciferase gene by qPCR. Reduced expression of EWS/FLI1 is always accompanied by a reduction of GLI1 and its direct target Patched1. Panel B: Similar qPCR results are obtained from other ESFT cell lines, A673 and CHLA9. Panel C: An unrelated antisense construct inhibits EWS/FLI1 expression by Western blot (left of panel). This reduction in EWS/FLI results similar alterations in the expression of GLI1 and Patched1 by real time qPCR (right of Panel C).

Our earlier findings demonstrated that GLI1 expression is dependent on EWS/FLI1 expression in one set of experimental conditions using in vivo EWS/FLI1 siRNA. If GLI1 upregulation by EWS/FLI1 is of biologic importance to ESFT, we would expect to find such a relationship applies to multiple means of targeting EWS/FLI1 and in multiple ESFT cell lines. Since off target effects can produce spurious results in RNAi experiments, we assessed the relationship between EWS/FLI1 and GLI1 activity in ESFT cell lines using several shRNA targeting sequences. In all cases, polyclonal cell lines were derived using lentivirally delivered shRNA followed by rapid selection in puromycin. As is seen in Figure 2A, the degree of EWS/FLI1 knockdown of expression mirrors the reduction in GLI1 and of Patched1 transcript. This close relationship is highly unlikely to have been produced by an off target effect. It also establishes Patched1 as a target of transcriptional modulation by EWS/FLI1 in ESFT cell lines.

Furthermore, similar polyclonal shRNA experiments have been shown to yield reductions of GLI1 and of Patched1 in both A673 and CHLA9 (see Figure 2B), indicating that the EWS/FLI1 dependence of GLI1 expression and signaling is shared among several well studied ESFT cell lines.

Finally, to further evaluate the soundness of this relationship, we employed an antisense form of EWS/FLI1 and selected stable knockdown clones from TC71 and TC32[15]. Figure 2C demonstrates once again that an experimentally induced reduction in EWS/FLI1 expression results in a reduction of GLI1 and Patched1 expression.

### Multiple means of GLI1 inhibition establish the importance of GLI1 expression to ESFT phenotype

**Figure 3:**
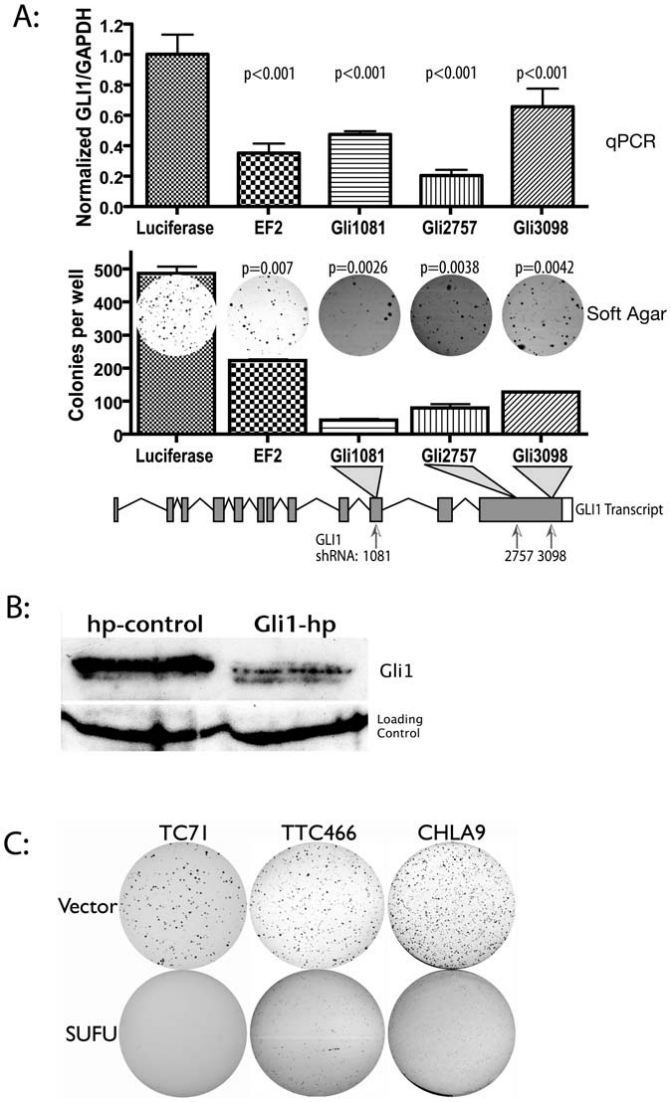
Multiple means of GLI inhibition, diminish ESFT cell line phenotype. Multiple GLI1 shRNA constructs (A, bottom) produce diminished GLI1 expression to varying degrees (Panel A, top) and diminished anchorage independent growth (Panel A, middle) in TC71. Panel B: GLI1 shRNA constructs produce the expected reduction in GLI1 protein by western blot in TC71. Panel C: Overexpression of the endogenous HH-GLI inhibitor Suppressor of Fused (SUFU) reduces anchorage independent growth of ESFT lines.

We have also demonstrated that GLI1 hairpin is capable of diminishing anchorage independent growth in ESFT cell lines. To rule out a possible off-target effect, we evaluated a panel of GLI1 shRNA targeting sequences in the ESFT cell line TC71. Figure 3A illustrates the targeting sequences used and depicts the degree of GLI1 expression decrease and the degree of decrease in anchorage independent growth. The consistent observation is that the degree of GLI1 knockdown is associated with a reduction in anchorage independent growth. Figure 3B demonstrates the efficacy of these constructs at reducing GLI1 protein levels. These findings suggest that GLI1 is clearly important to this aspect of tumor cell line phenotype and that this observation is unlikely to be due to an off target effect.

To further confirm the effect of HH-GLI inhibition in ESFT, we employed alternate means of inhibition. Suppressor of Fused (SUFU) is an endogenous inhibitor of the HH-GLI1 pathway acting by either cytoplasmic sequestration of GLI1 or by inhibition of GLI1 transcription at select loci[16], [17]. We drew upon our prior observation that overexpression of Suppressor of Fused (SUFU) is capable of diminishing anchorage independent growth of EWS/FLI1 transformed NIH3T3 cells[4]. To see if ESFT lines behave in a similar fashion, we transduced a total of three ESFT lines with SUFU under the control of a high activity retroviral promoter. High levels of SUFU transcript were exhibited by these polyclonal lines (data not shown). Figure 3C shows that all three lines exhibited diminished anchorage independent growth. These experiments were verified in 2–4 independent assays per line. They further strengthen the case that GLI1 dependence of ESFT lines is a widespread phenomenon.

### GLI1 signaling in ESFT is Hedgehog independent

**Figure 4:**
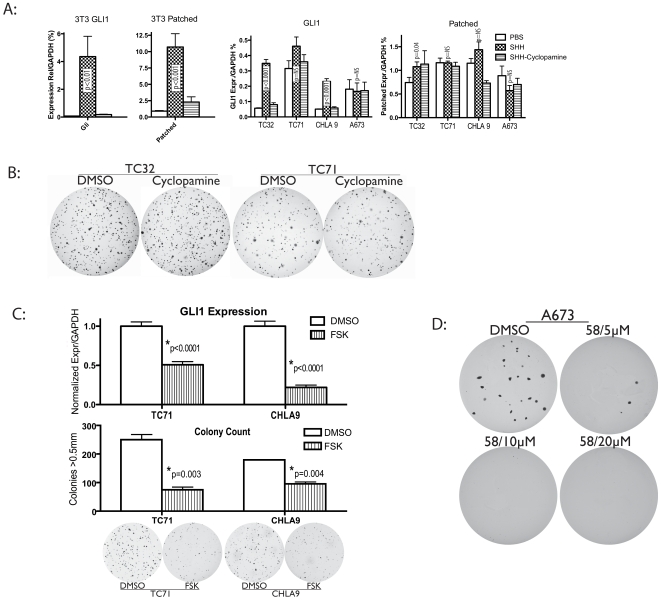
GLI1 upregulation is Hedgehog independent. Panel A: Exogenous SHH in culture media effectively upregulates GLI1 and Patched in NIH3T3, an SHH responsive cell line. Cyclopamine (5 mcM) effectively blocks this stimulation. However, the same human SHH protein is relatively ineffective in altering GLI1 expression in ESFT cell lines, with 2/4 lines showing no response. Patched1 expression is mildly altered (<50% change) in TC32, with 3/4 lines showing no change. Statistical comparisons are made with diluent. NS means “not significant.” Panel B: Consistent with the minimal expression changes produced by Cyclopamine, 10 mcM Cyclopamine is ineffective at altering anchorage independent growth in ESFT cell lines as was observed with GLI1 shRNA. Panel C: Forskolin, which blocks the pathway at the level of GLI1 at 100 mcM concentration, has similar inhibitory effects to those observed with shRNA and endogenous inhibitors. Panel D: Unlike Cyclopamine increasing concentrations of the specific GLI transcriptional inhibitor GANT58 results in abrogation of anchorage independent growth in the Ewing cell line A673.

The most common mechanism for activation of the HH-GLI pathway involves activation of Smoothened, either by overexpression of the HH ligand or by a loss of function mutation in the Patched1 tumor suppressor. In EWS/FLI1 transformed NIH3T3, there is evidence for only minimal HH activation[4]. Furthermore, expression data from primary Ewing tumor specimens does not demonstrate significant levels of HH ligand (Figure 1). To obtain further proof of the HH independence of GLI1 activation in ESFT, we assessed HH activation by the application of an active form of exogenous human SHH ligand to cells in culture. As shown on the left in Figure 4A, application of this ligand on the HH sensitive cell line NIH3T3 results in marked upregulation of GLI1 and of Patched1, demonstrating an intact HH-GLI transduction pathway with appropriate downstream signaling. The compound Cyclopamine, which blocks pathway activation at the level of Smoothened, effectively eliminates this activation as expected. In ESFT cell lines (Figure 4A, right), the picture is different. GLI1 transcript is upregulated by SHH to generally lesser degree, with 2/4 lines showing no response. While this increase in GLI1 transcript can be blocked by Cyclopamine, none of the cell lines demonstrate even a 50% enhancement of Patched1 with exogenous human SHH. So this lesser degree of GLI1 activation in ESFT cell lines is not accompanied by any major enhanced signaling downstream of GLI1. We conclude that, in addition to having little HH expression, ESFT cell lines are relatively HH insensitive. The consequence of this insensitivity is shown in Figure 4B as incubation of these lines with Cyclopamine produces little measurable effect on anchorage independent growth. These results contrast with the results presented above for GLI1 hairpin or overexpression of SUFU.

Cyclopamine acts on the HH-GLI pathway at the cell surface by inhibiting the activation of Smoothened[18]. The failure of Cyclopamine to recapitulate our shRNA and SUFU inhibitory studies suggest that GLI1 activation is a more central phenomenon is ESFT. To further confirm this impression we used another means of blockade. Forskolin (FSK) is a compound which effects cytoplasmic PKA stimulation and is known to inhibit the HH-GLI pathway at the level of GLI1[19], [20], [21]. While it is not a pure GLI1 inhibitor, it has been extensively used experimentally to block the pathway at this level [22], [23], [24], [25]. As is shown in Figure 4C, treatment of ESFT cells with FSK results in diminished expression of GLI1 transcript. As a consequence, anchorage independent growth is inhibited. This further suggests that GLI1 is important to ESFT biology and that it is activated downstream of Smoothened.

Finally, GANT58 (NSC75503) has been shown to inhibit transcriptional activation by GLI1 (as well as by the other GLI species) [9]. We exposed A673 cells to increasing concentrations of GANT58 and found a dramatic reduction in anchorage independent growth with concentrations as low as 5 mcM. This further supports a mechanism of activation which is downstream of Smoothened and is consistent with the recent observation of direct transcriptional activation of GLI1 by EWS/FLI1[5].

### Some GLI1 and EWS/FLI1 transcriptional targets overlap

GLI1 is an oncogenic transcription factor. If GLI1 is more than a peripheral event in ESFT biology, one might expect it to play a significant role in the transcriptional profile of EWS/FLI1 in ESFT. To evaluate this possibility, we assessed a published set of EWS/FLI1 targets[26]. Generated in A673 cells, this data set identified 31 genes upregulated by EWS/FLI1 as assessed by two separate shRNA hairpin sequences combined with data on restored expression of EWS/FLI1. In this target list, one finds GLI1. Also on this list are NKX2.2 and GAS1, two loci known to be affected by HH-GLI signaling. NKX2.2 is a Class II HH target in the developing neural tube[27]. Its expression is induced in neural ectoderm subsequent to SHH release by the notochord. More recently, it has been shown to be directly targeted by GLI1[28], much like Patched1. GAS1 is more indirectly involved as part of a feedback network of SHH binding factors. An enhancer of HH-GLI signaling, its expression is modulated based on the signaling conditions of the target tissue. Under conditions of low HH stimulation, GAS1 expression is enhanced; while in conditions of strong SHH signaling, its expression is downregulated[29]. Our own data indicate that the GLI1 target Patched1 is also transcriptionally upregulated by EWS/FLI1 in multiple ESFT lines (see Figure 2). The finding that several HH-GLI1 targets have also been identified EWS/FLI1 targets suggests a model in which EWS/FLI1 reaches these targets through GLI1.

**Figure 5:**
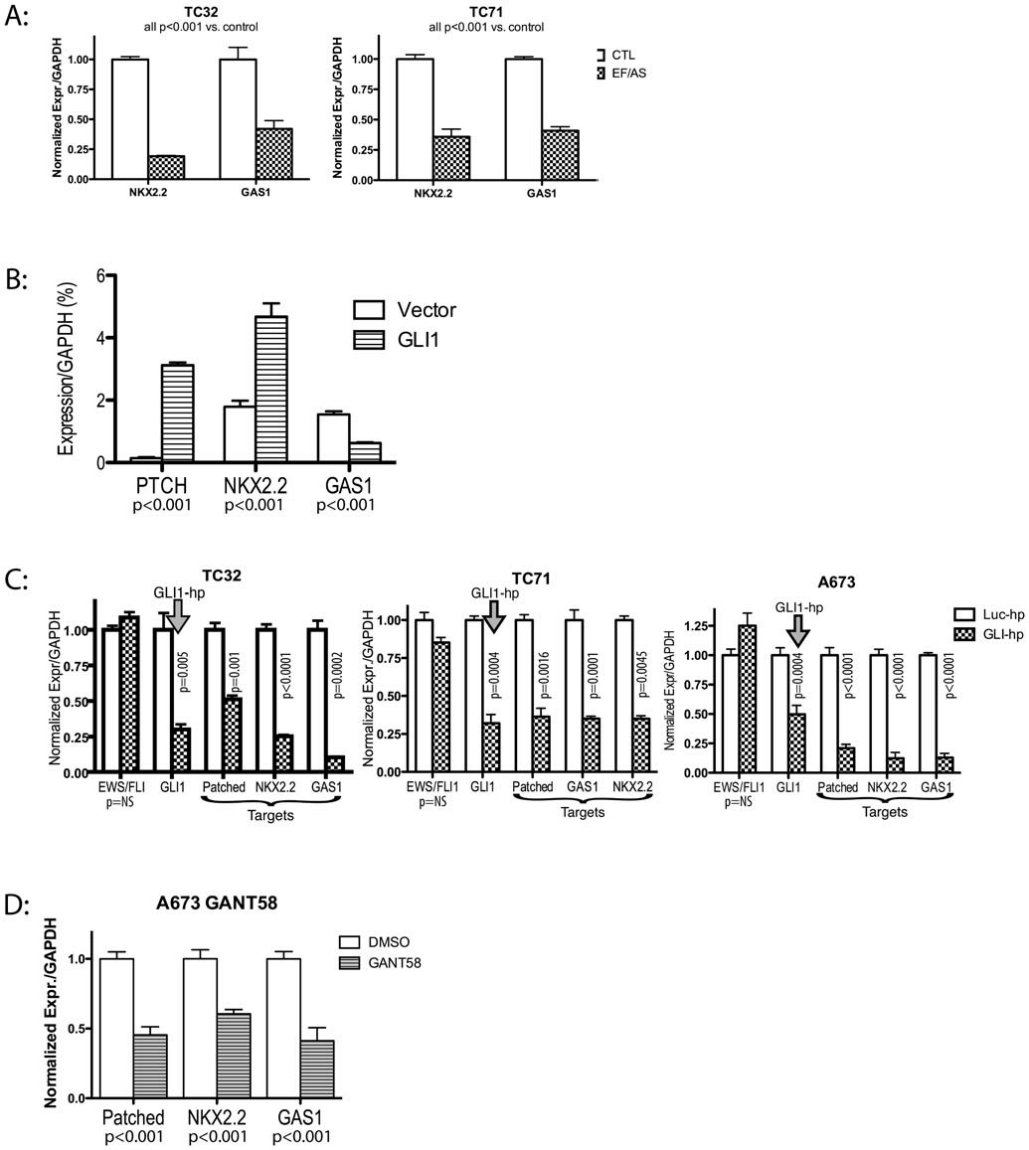
EWS/FLI1 and Gli1 targets overlap in ESFT. Panel A. EWS/FLI1 antisense (see Figure 1C) demonstrates that NKX2.2 and GAS1 are transcriptionally upregulated by EWS/FLI1 in TC71 and TC32. Panel B. GLI1 overexpression in TC32 shows that EWS/FLI1 targets PTCH, NKX2.2 and GAS1 are transcriptionally modulated by GLI1 expression in a Ewing cell background. Panel C. GLI1 shRNA reduces GLI1 expression and also reduces expression of these EWS/FLI1 targets. Panel D. GANT58 (10 mcM), an inhibitor of GLI transcriptional activation, results in transcriptional downregulation of our three putative downstream targets of GLI1.

To test this hypothesis, we first sought to demonstrate that NKX2.2 and GAS1 were also EWS/FLI1 modulated targets in TC32 and TC71, as has been shown in A673[26]. Figure 5A demonstrates that both are indeed transcriptionally upregulated by EWS/FLI1, based on data from cells transduced with EWS/FLI1 antisense. Next, we tested whether these EWS/FLI1-modulated targets are also GLI1 responsive in an ESFT background. Since TC32 cells have the lowest level of GLI1 expression of common ESFT cell lines[4], we overexpressed GLI1 in TC32 (Figure 5B). As direct GLI1 targets, Patched1 and NKX2.2 are upregulated by GLI1. This supraphysiologic GLI1 expression results in downregulation of GAS1, as has been observed with prolonged SHH stimulation in developmental models[29]. Nevertheless, the transcript levels of all three loci are clearly GLI1 modulated in a Ewing cell background.

As a further test that EWS/FLI1 targets these three loci through GLI1, we measured the effect of GLI1 inhibition via GLI1 shRNA. Figure 5C demonstrates that all three loci are transcriptionally downregulated by GLI1 hairpin, just as they diminish with EWS/FLI1 hairpin. The fact that GAS1 is downregulated by both GLI1 expression and by GLI1 knockdown reflects the different states of pathway activation between the more physiologic levels of GLI1 expression in the shRNA experiment and the dramatic overstimulation of the pathway with GLI1 overexpression.

As an additional proof of this model, we employed a different method of GLI1 transcriptional inhibition. The compound GANT58 has been shown to inhibit GLI transactivation [5], [9]. We have already shown that it has effects similar to GLI1 shRNA on anchorage independent growth in an ESFT line (see Figure 4D). If our model is correct, GANT58 should produce effects on downstream transcriptional targets similar to those seen with GLI1 shRNA. To test this, we exposed the ESFT cell line A673 to GANT58 at doses shown to inhibit GLI1 transactivation. Figure 5D demonstrates the anticipated transcriptional downregulation of Patched, NKX2.2 and GAS1 by treatment with GANT58.

## Discussion

Our findings demonstrate the widespread nature of EWS/FLI1 dependent GLI1 deregulation in ESFT cell lines. Deregulated expression of GLI1 is a characteristic of primary ESFT and many ESFT cell lines. GLI1 expression is important to support the malignant phenotype of ESFT cell lines whether inhibited by shRNA, endogenous regulators, or by pharmacologic agents. This deregulation appears to be Hedgehog independent. Finally, we have identified the first members of a subset of EWS/FLI1 targets which require GLI1 for their deregulation. The significant biologic effects of GLI1 inhibition in ESFT cell lines is not surprising, since inhibition of NKX2.2 alone is sufficient to produce similar significant effects[26]. The biologic effects of altered expression of our other two overlapping targets, Patched1 and GAS1 remain to be elucidated in ESFT.

As we have observed in a model systems [4] and has been seen in other ESFT cell lines [5], EWS/FLI1 deregulation of GLI1 appears to the a Hedgehog independent phenomenon. The mechanism by which GLI1 is upregulated has been recently described as being direct transcriptional upregulation by EWS/FLI1[5]. It is gratifying that data from this publication agrees with our hypothesis of activation of GLI1 by a Hedgehog independent mechanism downstream of Smoothened. These observations will enable a broader understanding of the range of activity of EWS/FLI1 and will add to the growing literature on non-canonical mechanisms of activating HH-GLI signaling[30].

With the recent opportunities afforded by microarray analysis, a more consistent set of EWS/FLI1 targets is emerging[3]. Such target sets are biased by the means by which they are identified. As such, these methods are likely to favor targets whose transcript levels are inherently high, such as those which may be strong direct EWS/FLI1 transcriptional targets [31]. However, there is no reason to assume that the list of biologically significant EWS/FLI1 targets must necessarily be restricted to the “high amplitude” set favored by microarray analysis.

Given the literature suggesting that Patched1, NKX2.2, and GAS1 are components of the HH-GLI signaling network, the most likely model is for EWS/FLI1 induced elevation of GLI1 expression leading to altered expression of a number of target loci. Further characterization of this system has the potential to provide some order to the complex picture of transcriptional deregulation by EWS/FLI1. While it is likely that only a subset of EWS/FLI1 targets are deregulated through GLI1, the data presented here and the known biology of HH-GLI signaling suggest that many may be of biologic import. Since GLI1 acts as a transcription factor, analysis of shRNA-manipulated ESFT cell lines by expression microarray would allow the identification of common targets of EWS/FLI1 and GLI1. Knowledge of such a hierarchy can be of tremendous benefit in the design of new therapies. Certainly, targeting an EWS/FLI1 mediator which is more upstream, such as GLI1, could be expected to have more extensive effects than targeting a gene further downstream in the process. Our work with the GLI1 inhibitor GANT58 suggests that this pathway may be a valid target for translational research in Ewing Sarcoma. A secondary benefit of employing GLI1 inhibition in Ewing tumors is that GLI1, unlike EWS/FLI1, is felt to be involved in the pathogenesis of a large number of common malignancies. As market-driven forces generate means of targeting this pathway in common adult malignancies[8], the potential application of these means to patients with Ewing Sarcoma Family Tumors promises to improve outcomes in this difficult malignancy.

## Materials and Methods

### Retroviral Experiments

The generation of recombinant retroviral stocks has been previously described [32] and in these experiments was modified only by using the LINX-A packaging line (Genetica) for retroviral constructs. These experiments employed either the vector system in that description or pLXIN (Clontech, Mountain View, CA) or the derivative pLXIH[4]. The retroviral construct for murine SHH was previously described [4]. The retroviral construct for Human Suppressor of Fused was obtained by PCR amplification of the reading frame from the clone MGC3533158 (Invitrogen). This insert was cloned into pLXIN (Clontech) or pLXIH [4]. Cloning strategy is available on request.

### RNAi experiments

shRNA experiments were performed as previously described[4]. Target oligo sequences were obtained from the following publications: EF2 [26], EF4 [26], EF818 [33]. Other shRNA oligo sequences can be found in Figure S1 accompanying this paper online.

The antisense EWS/FLI1 construct and its use has been previously described [15].

### Real Time Quantitative PCR (qPCR)

RNA was isolated using either Tri-Reagent (MRC) or RNeasy Plus Kit (Qiagen, Valencia, CA) according to manufacturers instructions. CDNA's were prepared from 0.5 mcg total RNA using the BioRad iScript Kit according to manufacturer's instructions. PCR was performed using a BioRad MyiQ Thermal Cycler using BioRad iQ SYBR Green Supermix according to manufacturer instructions (BioRad, Hercules, CA). Conditions selected were 30 seconds of denaturation at 94°C and annealing/extension for one minute at 60°C. Data was analyzed for expression relative to GAPDH using the comparative C_t_ method with data resulting from the average of three replica wells within each experiment. Data in figures is represented either as a percentage of GAPDH expression or the percent GAPDH values have been normalized with the control value set at 1.0. Results shown average the results of three independent experiments. Error bars show the standard error of the mean. P values were calculated using an unpaired Student's t-Test. Primers for human GLI1, GLI2, Patched1, and GAPDH have been previously described[4]. Other primers sequences are in Figure S1 online.

### Cells, Cell Culture, and Materials

All tumor cell lines were grown in RPMI supplemented with 2 mM glutamine and 10% Fetal Bovine Serum at 37 degrees C and in 5% CO_2_. TC71, TC32, TTC466, and TTC475 (plus primary specimen) were kindly provided by Dr. Tim Triche. CHLA9 was provided by Dr. C. Patrick Reynolds. A673 cells were obtained directly from ATCC.

Primary tumor specimens were obtained from Cooperative Human Tissue Network (CHTN) (http://www.chtn.nci.nih.gov). RMS-R and RMS-T RNA specimens were kindly provided by Dr. Michael Anderson. SaOS RNA was kindly provided by Dr. Daniel Wai. We appreciate their help in this regard.

### Drug Experiments

Cells were exposed to Sonic Hedgehog C24II amino-terminal peptide (R&D Systems: http://www.rndsystems.com) for 16 hours before RNA was extracted.

GANT58 and Forskolin (FSK) were obtained from EMD Biosciences (http://www.emdbiosciences.com, Cat #344270). Cyclopamine was obtained from Toronto Research Chemicals (http://www.trc-canada.com). Drugs were dissolved in DMSO. For expression experiments, cells were incubated with cells overnight.

### Antibodies and Immunoblotting

These were performed as previously described[4].

### Soft Agar Transformation Assay

Polyclonal, selected populations of the cell type to be analyzed were plated in soft agar at either 5000 or 15000 cells per well of a 6-well plate. The agar was made with Iscove's medium containing 20% fetal calf serum for tumor cell lines as described previously [32]. Drugs or diluent were added to the top layer of the agar in an amount to achieve the target concentration in the whole well. Drug was added only at the time of Agar setup. Agar plates were imaged via a transilluminating flatbed scanner approximately 2–3 weeks after plating. Counts were performed manually on high resolution scanned images. Results shown for these agar assays were consistent over at least three independent experiments. Lighter background color on some scanned agar images results from acidification of phenol red in the media, a sign of enhanced anchorage independent growth.

### Reproducibility

Data presented has been repeated at least three times with consistent results. Numerical data presented is the average of these replicated experiments. Where necessary, results across experiments have been normalized to a control value. Error bars presented are the standard error of the mean.

## Supporting Information

Figure S1 Oligonucleotide sequences
(0.00 MB RTF)
