# Supplementary material for: GLI1 Is a Central Mediator of EWS/FLI1 Signaling in Ewing Tumors
Source: PLoS One. 2009 Oct 27;4(10):e7608. doi: 10.1371/journal.pone.0007608 (PMC2763206; doi:10.1371/journal.pone.0007608)
Supplement: Figure S1 — Oligonucleotide sequences (0.00 MB RTF) [file pone.0007608.s001.rtf]

Figure S1: Joo, et al., GLI1 is a central mediator of EWS/FLI1 signaling in Ewing TumorsshRNA oligo sequences used (in pSIF or pFIV vector, System Biosciences)EF-bpFW: GATC C GGCAGCAGAACCCTTCTTA TTCAAGAGA TAAGAAGGGTTCTGCTGCC TTTTTGRV: AATTCAAAAA GGCAGCAGAACCCTTCTTA TCTCTTGAA TAAGAAGGGTTCTGCTGCC GGli1-1081FW: GATCCG CGAGGGCTGCAGTAAAGCC TTCAAGAGA GGCTTTACTGCAGCCCTCG TTTTTGRV: AATTCAAAAA CGAGGGCTGCAGTAAAGCC TCTCTTGAA GGCTTTACTGCAGCCCTCG CGGli1-2757 FW: GATCCG GGCTCAGCTTGTGTGTAAT TTCAAGAGA ATTACACACAAGCTGAGCC TTTTTGRV: AATTCAAAAA GGCTCAGCTTGTGTGTAAT TCTCTTGAA ATTACACACAAGCTGAGCC CGGli1-3098FW: GATCCG ACCCCAGCTGTGGTCATCC TTCAAGAGA GGATGACCACAGCTGGGGT TTTTTGRV: AATTCAAAAA ACCCCAGCTGTGGTCATCC TCTCTTGAA GGATGACCACAGCTGGGGT CGQuantitative PCR primer pairs (given as sense//antisense, all 5’-3’)As cited in Zwerner JP, et al., Oncogene 27: 3282-3291--included here as a convenience:Human GLI1: GAACCTTCCTACCAGAGTCC//GTGCTGCTGCCCTATGTGHuman GLI1-ALT: CTTGTGGTCCCCATGACTCT//GATTCAGGCTCACGCTTCTCHuman GLI2: AGATGTTGTAAGAGAAGGTTTATG//CGTTAGCCGAATGTCAGCHuman PATCHED1: ACAAACTCCTGGTGCAAACC//CTTTGTCGTGGACCCATTCTHuman GAPDH: TCCTCTGACTTCAACAGCGACA//ATGGTACATGACAAGGTGCGGNewly described primers:Human SHH: TTAGCCTACAAGCAGTTTATCC//TCCTTACACCTCTGAGTCATCHuman IHH: GCTATCTCGGTGATGAAC//CAGCAGTCCATACTTATTGHuman BMI1: CGTGTATTGTTCGTTACCTGGAGA//ATCACAGTCATTGCTGCTGGGCHuman NKX2.2: GAACCCCTTCTACGACAGCA//GGGTCTCCTTGTCATTGTCCHuman GAS1: GAAGGGATGGTTGGGGATAC//GCAGACGAGTTGGGAGTTTC
